# Evaluation of the Sealing Ability of Amalgam, MTA, Portland Cement and Coltozol in the Repair of Furcal Perforations

**Published:** 2006-07-01

**Authors:** Zohreh Ahangari, Mahdieh Karami

**Affiliations:** 1 *Department of Endodontics, Dental School, Shahid Beheshti University of Medical Sciences, Tehran, Iran*; 2 *Member of Shahid Kalantari Clinic, Tehran, Iran*

**Keywords:** Furcation, Perforations, Repair

## Abstract

**INTRODUCTION:** Perforations of the pulpal floor create problems during endodontic treatment and often results in secondary periodontal involvement with eventual loss of teeth. The purpose of this study was to evaluate the ability of mineral trioxide aggregate (MTA), Amalgam, zinc oxide and eugenol (ZOE) and Portland cement to seal furcal perforations in extracted human molars using dye penetration.

**MATERIALS AND METHODS:** This Microleakage study was conducted on 70 human permanent mandibular molars with well developed, non-fused roots. Access openings and furcation perforations were prepared in the pulp chamber floor. Ten teeth were assigned as controls and divided into two groups of five teeth each as positive and negative control. The positive group were accessed and perforated similar to experimental groups but the negative controls were not prepared. The rest of the teeth were divided in four experimental groups of 15 teeth in each group. Experimental groups comprised groups Al to A4, group Al was repaired with MTA, group A2 with Amalgam, group A3 with Coltozol (ZOE) and group A4 was repaired with Portland cement. The teeth were submerged in solution of 2% fucshin dye for 24 hours. Finally, the samples were sectioned and evaluated for linear dye leakage at X25 magnification and analyzed statistically.

**RESULTS:** The results showed that MTA had significantly less leakage than Amalgam, Portland cement and Coltozol (P<0.05).

**CONCLUSION:** Maximum dye penetration was observed in Coltozol followed in decreasing order by Portland cement and Amalgam.

## INTRODUCTION

Endodontic therapy is the treatment of pulp and radicular pathosis and aims to eliminate all bacteria and destructive factors that can lead to defects like abscess and bone infection. Complicated problems may be encountered during root canal treatment. There may be a need for periradicular surgery, sealing perforations (with or without surgery) and procedures like direct pulp capping (DPC) and apexification (1).

Perforations in the crown, floor of the pulp chamber or 1/3 of the coronal roots are errors that occur when care is not taken during the creation of a favorable access opening. Furcation perforations are one of the complications which may be encountered during endodontic therapy .Immediate sealing perforations with suitable materials lead to successful treatment. Therefore an accessible, economical and effective, biocompatible sealing material is necessary.

Ingle in the Washington study which is the most valid study in the field of investigating success and failure of endodontic treatments in 1961, reported that 9.61% of failures was due to perforations which occur when trying to find the orifice of the canals in the floor of the pulp chamber or during post preparation (2). Also excessive widening of the coronal part of curved roots will result in lateral perforation of root canals and therefore inflammatory response in the periodontium. In order to treat the perforation nonsurgical repair was preferred to surgery (3). Delay in restoring perforations, will eventually lead to losing the teeth because of deep pockets (4).

In order to gain a good prognosis the material should be sealable, biocompatible (non toxic), not polluted with blood, no extrusion of repair material through perforation during condensation, induce bone formation and healing, radiopaque, induce mineralization and cementogenesis (5-12).

Numerous studies relate to the repair of perforations, namely sealing ability of MTA, Amalgam and IRM in 50 mandibular molars with lateral perforations performed in 1993 (3). In this study MTA had the least leakage because of being hydrophilic, but no significant difference was found between restorations with Amalgam and IRM.

Also another study describing the sealing ability of Amalgam, Cavit and light cured glass ionomer on 40 mandibular and maxillary molar perforations showed that Amalgam had the most leakage but Cavit stood in the next place and light cured glass ionomer had the least leakage because of the ability of glass ionomer to bond to dentin (13). During setting, calcium ions of the dental hard tissue form a chemical bridge between tooth structure and the glass ionomer. In addition, light cured glass ionomer cement had a flow property that is an advantage in sealing the apical end of the perforation.

The aim of this study was to compare the sealing ability of mineral trioxide aggregate (MTA), Amalgam, zinc oxide and eugenol (ZOE) and Portland cement, when used to repair the perforations created in furcation area of extracted molars.

## MATERIALS AND METHODS

Seventy extracted mandibular molars were used in this study. The teeth were caries free with nonfused well-developed roots. After extracting the teeth they were kept in sodium hypochlorite %5 for 30 minutes. They were then cleaned of calculus, soft tissue tags, debris and attached bone by a periodontal curette and washed with tap water and kept in normal saline until next step.

First a standard access was prepared on the occlusal surface of the teeth in experimental group with the use of a diamond bur in a high­ speed handpiece along with water spray. Alginate impression was subsequently taken from every group of teeth as it would serve as a jaw for the teeth, similar to a stop or barrier when condensing the repair material into the perforation area. In the next step a perforation was made in the centre of the floor of the pulp chamber of the teeth along an axis parallel to the long axis of the teeth, while holding the teeth in our hands. The bur was renewed after every five perforation made in the centre of the pulp chamber. The width of the perforations was equal to the diameter of the perforating bur, but the length depended on the dentin­ cementum thickness. However the perforation proceeded to 5mm of the file #80 length. Then they were washed with water and dried in air. Moist cotton pellets were passively placed between the roots in the furcation areas during repair of the perforations in order to represent the oral cavity without any role as a barrier or matrix. The teeth were placed in the Alginate impression taken earlier.

Sixty teeth were randomly placed in four groups of 15 teeth each and designated A1, A2, A3 and A4. The remaining 10 from 70 teeth were divided into two positive and negative control groups of 5 teeth each.

Perforation areas restored with MTA, Portland cement, Amalgam and Coltozol were designated as groups A1 to A4 respectively.

The repair material were placed with a messing gunpack on the perforated areas and packed with a condenser followed by the access cavity sealed with Coltozol, which also sealed the coronal area. The teeth were placed in a thermocycling device for 2 days (5 cycles) in order to simulate the oral cavity.

Having removed the teeth from the device, they were covered with 2 layers of nail polish of 1 to 2 mm except around the perforated area, such that the dye would only penetrate through the furcation area, the dentinal tubules or lateral canals. Also the teeth in the negative control group which were not prepared were totally covered with 2 layers of nail polish, in order to demonstrate that the nail polish was a good barrier against dye penetration. The five teeth in the positive control group were accessed and perforated in the same manner as those in experimental groups without any further treatment. The main objective was to demonstrate that the dye used in this study could penetrate through the perforation.

All the teeth in the four experimental groups (A1-A4) and two control groups (B1-B2) were then immersed in 2% fucshin for 24 hours at room temperature. After removal from the solution and in order to get ready for sectioning, the teeth were washed with tap water and every sample was placed separately in a transparent acryl impression. The teeth were sectioned mesiodistally parallel to the long axis of the teeth exactly in the middle of the perforation area. The mesial and distal walls of the sections were evaluated with the use of a stereomicroscope (x25) and a scale graded in hundreds of a millimeter. Leakage was measured on each wall, as the amount of linear dye penetration from the apical end of the perforation to the pulp chamber floor.

Raw data were recorded and analyzed for Statistical significance using analysis of variance (ANOVA). Statistical analysis to be described at a level of significance of p=0.05 according to mean, average, standard deviation.

## RESULTS

In the present study, the positive control teeth showed complete dye penetration throughout both the perforation area and access preparation. The negative controls did not demonstrate any dye penetration.

The least amount of leakage was seen in the teeth restored with MTA but it was the highest in the group repaired with Coltozol. In the experimental groups, all materials revealed different degrees of microleakage (Table 1) which was described. The discrepancy of mean micro leakage between each group and other repairing materials was evaluated by the statistical test of least square differences.

Where, the median micro leakage discrepancy between samples filled with MTA was compared to samples restored with Portland cement, Amalgam and Coltozol which were 0.532 mm, 0.271mm and 0.779 mm respectively. The difference in the case of Portland cement and Coltozol was statistically significant (P<0.01). Furthermore, the difference was also significant with respect to Amalgam restorations (P<0.05).

In the group of Portland cement restorations, the difference of median micro leakage discrepancy with Amalgam and Coltozol were 0.261 and 0.247mm respectively which were not significant. But significant difference (0.508 mm) was formed between Amalgam and Coltozol (P<0.01).

## DISCUSSION

The prognosis of endodontically treated teeth becomes worse when root perforations occur because of the damage to the periodontal attachment apparatus. Sealing the perforation is therefore of paramount importance for healing to occur. Moisture, bleeding, unconventional accessibility, and a bottomless cavity, however make repair of the perforation difficult which will eventually have a great impact on the prognosis of the perforated teeth.

The present study describes the ability of MTA, Amalgam, Portland cement and Coltozol in sealing the perforations of the floor of pulp chamber in 70 mandibular molars. Many studies have been conducted on evaluating the ability of different materials in repairing clinical defects.

In our study MTA had significantly the least Microleakage compared with Amalgam, Portland cement and Coltozol. Lee and Torabinejad in 1993 compared MTA with Amalgam and IRM in repair of furcation perforations and found that MTA was the best (3).

Nakata (14) in 1998 compared Amalgam with MTA and concluded that MTA had the least micro leakage in restoring furcation perforations. Daoudi (4) in 2002 investigated usage of endodontic microscope in different samples such as MTA, vitre bond. He discovered that MTA had the least micro leakage.

**Table 1:** LSD between groups

**Group**	**Second Group**	**M.** **Difference**	**P-value**
MTA	Portland	0.532	0.000
	Amalgam	0.271	0.051
	Coltozol	0.779	0.000
Portland	Amalgam	0.261	0.060
	Coltozol	0.247	0.075
Amalgam	Coltozol	0.508	0.000

The results of these studies were in Concordance with our study that the least micro leakage seen in restoring furcation areas was repairing with MTA. A possible explanation could be that the main part of MTA was mineral oxide, which for setting it had to react with water. It was hyrodophilic and the moisture in the surrounding tissue activated the chemical reactions. Therefore moisture was importance when using MTA (3). Mixing the powder with water turns it into a colloidal gel which sets in four hours. The characteristics of the mixture depended on the size of the particles, the ratio of powder to water, heat and presence of water.

In many studies Amalgam was recommended as a restoring material for furcation problems (15). However according to the results it had not been successful because in case of furcation perforation, the defect was bottomless, and Amalgam cannot be well condensed into the perforation in order to provide the desired seal. Well-condensed Amalgam adapted better to the cavity walls and provided a homogenous filling with a relatively good seal (9).

In our study the mean micro leakage for Amalgam was 0.496mm, which compared with MTA was statistically significant (P<0.01). The results in the studies of Lee (3) in 1993 and Nakata (14) in 1998, confirmed the findings in relation to Amalgam. The physical and chemical characteristics of MTA and Amalgam are the main reasons for our findings.

In the present study we did not assess overfill and underfill cases in the perforated areas. Benenati (16) demonstrated that 70% of failures in furcation perforation restorations are because of overfill. As Amalgam had to be well condensed into the perforation area (14) it could not provide a relatively good seal compared with MTA.

In Alhadainy's study in 1993 microscopic examination of the repaired perforations demonstrated that Amalgam was a nonhomogeneous, poorly condensed mass (5).

In our study the median micro leakage for Coltozol was 1.004 mm which had a significant statistical difference with MTA, Amalgam (P<0.001) and also had the highest amount of dye penetration compared with MTA, Amalgam and Portland cement. ELDeeb (17) in his study found that Cavit was suitable for restoring furcation perforations next to Amalgam and had the highest amount of micro leakage compared with other materials.

Another material investigated in our study was Portland cement for which there was no published report or information regarding the amount of micro leakage. However the use of Portland cement was more economical 75% of its chemical composition was similar to MTA, we therefore decided to investigate Portland type five as a repair material in restoring furcation perforations. According to our findings, the mean micro leakage of this material was 0.757 mm which compared with MTA was statistically significant. Although these two materials have the same composition, nevertheless, in practice the greater size of particles in the Portland cement and also the rapid loss of water and dehydration in this cement, made it difficult to condense the material into the perforation in practice.

So greater particle size and rapid water loss in Portland cement creates a gap between the restoring material and tooth wall, where dye could penetrate easily. However, it seems that dehydration was prevented if the ratio of water to cement (W/C) was increased .According to some studies on Portland cement could be strengthened and by adding limestone as filler and also by increasing the amount of the filler. Therefore, durability would increase by filling the voids by limestone and also reducing W/C (18). It seemed that MTA was also a type of Portland cement with smaller particles of limestone as filler.

The Microleakage of Portland cement type 5 with and without limestone filler is recommended for another study.

## CONCLUSION

The results of the present study demonstrated that MTA had significantly lower micro leakage compared with Amalgam, Coltozol and Portland cement (P<0.05). The highest amount of dye penetration was found in Coltozol and in decreasing order in Portland cement and Amalgam.
